# Supplementary material for: Collaborative intelligence in AI: Evaluating the performance of a council of AIs on the USMLE
Source: PLOS Digit Health. 2025 Oct 9;4(10):e0000787. doi: 10.1371/journal.pdig.0000787 (PMC12510544; doi:10.1371/journal.pdig.0000787)
Supplement: S2 File — (ZIP) [file pdig.0000787.s002.zip › S2 Data Raw data files containing unprocessed question inputs and LLM outputs including transcripts of deliberations/STEP3_Question-119-1_Type-MC-NJ.docx]

**Facilitator** **[Round 1]:** A 55-year-old woman, gravida 1, para 1, comes to the office because she is concerned that she has continued to have regular menses while her friends of the same age have gone through menopause. She reports having her usual premenstrual breast tenderness and menstrual cramping. She has not had change in flow, intermenstrual spotting, hot flushes, or night sweats. Medical history is unremarkable. She takes no medications. She does not smoke cigarettes. She drinks one glass of wine each evening. Her last menstrual period occurred 3 weeks ago. Screening mammogram obtained 1 year ago and all previous Pap smears, including her most recent Pap smear 2 years ago, have shown no abnormalities. Her 50-year-old sister also has not gone through menopause. The patient does not exercise. She is 157 cm (5 ft 2 in) tall and weighs 80 kg (176 lb); BMI is 32 kg/m². Vital signs are normal. Physical examination discloses adiposity of the abdomen. Pelvic examination discloses pink and rugose vaginal walls and a small uterus. The remainder of the physical examination discloses no abnormalities. Which of the following is the most appropriate next step to address the patient's continued menstruation? (A) Order fine-needle biopsy of the endometrium (B) Order pelvic ultrasonography (C) Order serum estradiol and follicle-stimulating hormone concentrations (D) Provide reassurance that she is simply still premenopausal (E) Recommend losing weight to help facilitate the onset of menopause (F) Recommend monthly leuprolide acetate injections for a 3-month duration (G) Refer the patient to an endocrinologist

**AI_A:** RESTATING THE QUESTION: This 55-year-old woman manifests continued menstruation, normal menstrual symptoms, and no menopausal symptoms, despite her peers experiencing menopause. She doesn't take any medication, doesn't smoke, drinks moderately, and her last menstrual period was three weeks ago. Family history suggests late onset of menopause. She's overweight and displays abdominal adiposity. All tests, including mammography and Pap smears, are normal. After examining her abdomen and pelvic region, the main concern is determining the most appropriate step forward, taking into consideration her continuous menstruation. PLANNING: Possible considerations for her continuous menstruation might include late menopause onset, which is not unusual and can be hereditary, or a hormonal imbalance that could affect the menstrual cycle. However, her physical exam and symptoms do not suggest malignancy, fibroids, or other anatomic causes. Therefore, we may need to consider doing hormone level tests, consulting an endocrinologist, or reassurance that she is still in her premenopausal phase. CLINICAL REASONING: If this was a case of anatomic abnormalities or endometrial malignancy, pelvic pain, abnormal bleeding, or changes in menstrual flow would be expected. Also, a significantly enlarged uterus is typically present with fibroids. However, none of these signs are present. The hormone levels can give some insights, but without common menopausal signs like hot flushes and night sweats, the likelihood of hormonal imbalance is less. The absence of these signs and symptoms also makes the referral to an endocrinologist unwarranted. Lastly, hereditary factors can influence the timing of menopause, and her sister's late menopause as well enhances this assumption. CONCLUDING: Weighing all these factors, it seems that this patient is most likely just still in her premenopausal phase. There doesn't seem to be immediate medical concern that would necessitate tests or interventions. Thus, patient reassurance might just be the most appropriate further step. RESPONSE: Therefore, the appropriate next step to address the patient's continued menstruation would be to provide reassurance that she is simply still premenopausal (D), taking into account the absence of menopausal signs and the presence of regular menses. This seems to be a case of late-onset menopause, possibly influenced by hereditary factors, given that her sister also has not gone through menopause at 50 years old.

**AI_B:** RESTATING THE QUESTION: Our patient is a 55-year-old woman who is concerned with her continued regular menstruation despite her age, compared to her peers who have already experienced menopause. Menopause, defined as the termination of the menstrual cycle typically around the age of 51, is characterized by hormonal changes including a decrease in estradiol and an increase in follicle-stimulating hormone (FSH). Our patient's presentation does not show signs of menopause as there are no changes in menstruation and she does not experience symptoms such as hot flashes or night sweats. Her familial history of late menopause (her 50-year-old sister) could be indicative of genetic factors influencing her reproductive timeline. Considering her symptoms and findings, we need to decide on the most appropriate subsequent step for this patient. PLANNING: The fundamental assumption here could be the patient is simply experiencing a late menopause. This assumption can be questioned due to the patient's older age and potential risk factors, such as her obesity (BMI of 32 kg/m²). The prioritized hypothesis could be hormonal imbalances delayed her menopause, or alternatively, she has an underlying pathological condition delaying menopause. CLINICAL REASONING: In determining what to do next, we look at potential causes. If she naturally has not yet experienced menopause, routine lab tests like serum estradiol and FSH can help establish the baseline hormonal levels and periods, and we can continue to monitor her. However, considering her age and obesity, the risk of endometrial hyperplasia and carcinoma increases, both of which could contribute to continued menstruation. Abdominal obesity, with adiposity observed in our patient's physical examination, is known to increase estrogen levels, hence potentially influencing menopause. CONCLUDING: Although there's no evidence of an obvious pathology, given her age and risk factors, we should lean towards more prudent investigations rather than mere reassurance or lifestyle modification advice. Targeted medical tests need to be done to clarify any potential imbalances or risks. These include hormonal assays and imaging studies. RESPONSE: I recommend ordering serum estradiol and follicle-stimulating hormone concentrations (Option C), along with pelvic ultrasonography (Option B) to rule out any underlying endometrial pathology.

**AI_D:** RESTATING THE QUESTION: A woman of 55 years old, who has had one pregnancy and given birth once, is concerned that she's still menstruating normally even though her peers have already experienced menopause. Her uterus is small, vaginal walls appear normal, and there have been no abnormalities in her Pap smears or mammograms. Her physical health otherwise seems to reveal no concerning signs. She is overweight. Her sister who is five years younger hasn't gone through menopause either. She is concerned about the fact she hasn't reached menopause yet and is looking for appropriate secondary steps. The options include various medical interventions as well as specific advice and or lifestyle changes. PLANNING: Assumption 1: The woman's delayed menopause is due to a pathological condition and requires a medical intervention. Assumption 2: The delay in menopause is due to her lifestyle and obesity. Assumption 3: The woman's delayed menopause is a normal physiological variation within the spectrum of healthy human conditions. This might be supported by the indication that the patient's younger sister also hasn't experienced menopause. After questioning and evaluating, my hypothesis would lean towards Assumption 3 as the one to prioritize clinically, as the patient doesn't display other symptomatic red flags often associated with pathologies related to menopause or endocrine issues. CLINICAL REASONING: Normal menopausal age varies from 45 to 55 years. The fact that this patient is 55 and not experiencing menopause is not intrinsically concerning in itself. This woman doesn't exhibit signs of abnormal uterine bleeding or menopausal symptoms such as hot flashes or night sweats, which would suggest a need for a more serious clinical intervention. Additionally, her sister's similar menstrual pattern suggests a possible genetic tendency towards later menopause. These factors suggest her body is still producing hormone levels consistent with those needed to maintain the menstrual cycle. Moreover, obesity can lead to increased estrogen levels, potentially delaying menopause. Thus, even though obesity can have negative health impacts, and weight loss might be recommended for overall health improvement, it's not necessarily linked to inducing menopause in this case. CONCLUDING: Given all this, it seems reasonable to assume that this patient is simply still within the normal range of age for the onset of menopause and her continued menstruation does not warrant invasive procedures or hormonal tests. RESPONSE: (D) Provide reassurance that she is simply still premenopausal.

**AI_C:** 1) RESTATING THE QUESTION: A 55-year-old woman who has been pregnant once and gave birth once, is concerned that her menstrual cycles persist while her peers' have stopped due to menopause. Despite her age, she exhibits no symptoms that are typically associated with the perimenopausal phase such as hot flushes, night sweats, or irregular menses. She maintains a lifestyle with moderate alcohol consumption, no smoking, and minimal physical activities. Further, she presents characteristics of obesity, which is a contributing factor to the estrogen production in the body. Her reproductive organs appear normal upon physical examination. 2) PLANNING: Given the patient's age, irregular or cessation in menstrual cycle could indicate the onset of menopause. However, the presence of regular menses and absence of perimenopausal symptoms could indicate that she is still in the premenopausal phase. Evidence-based guidelines state that the mean age of menopause is 51, with most women reaching menopause between the ages of 45 and 55. Some women can reach menopause later, especially if there is a family history of late menopause. A key point here is the patient’s sister who is 50 and hasn't reached menopause either. Thus, it's crucial to determine the patient's menopausal status to inform the next clinical steps. 3) CLINICAL REASONING: Before considering interventional steps such as endometrial biopsy, ultrasonography, weight loss, hormonal treatment, or referral to an endocrinologist, a reasonable starting point is to obtain crucial hormonal parameters. If this patient is nearing menopause or in early menopause, we would expect to see high levels of follicle-stimulating hormone (FSH) and low levels of estradiol due to the decreased function of the ovaries. Also considering the fact that BMI is 32, some of the estrogen activity might be maintained by peripheral conversion of androgens to estrogens in adipose tissue. On the other hand, if she is still premenopausal, we would anticipate FSH and estradiol levels reflecting a normal menstrual cycle. The consequent steps will be determined based on those test results, as it will provide us with more context about her hormonal status. 4) CONCLUDING: Understanding the woman's hormonal status is fundamental here. This provides the groundwork for delineating whether her continued menses is normal due to late menopause or a pathological process indicative of an underlying condition. Moreover, the obtained hormonal levels would provide the patient with an evidence-based reassurance or necessary intervention steps. 5) RESPONSE: The most appropriate next step in this case would be to (C) Order serum estradiol and follicle-stimulating hormone concentrations. Through this, we can determine her hormonal status and whether she is in fact premenopausal or nearing the onset of menopause. Armed with that information it would be easier to decide what the next step should be, or simply provide her with reassurances if all the results come back as normal for her age.

**AI_E:** 1) RESTATING THE QUESTION: The question concerns a 55-year-old woman who is puzzled as to why she is still menstruating when most of her friends of the same age have gone through menopause. She has not had symptoms often associated with menopause, like hot flushes or night sweats, and her menstrual flow has not changed. Her mammogram and Pap smears all show no abnormalities. The patient has never exercised, is overweight, and drinks a glass of wine each evening. Her sister, who is 50 years old, has also not experienced menopause. All her vital signs and physical examination are normal. The question seeks to determine what the best approach should be in addressing her continued menstruation. 2) PLANNING: Various assumptions could be made regarding this patient. One could assume that she might be experiencing abnormal uterine changes resulting in delayed menopause requiring further investigations like an endometrial biopsy or pelvic ultrasonography. Another assumption could be that she could be experiencing a hormonal problem warranting further endocrine evaluation or hormonal therapy. Yet we could alternatively propose she's simply premenopausal, given her sister's similar menopausal status. This situation isn't entirely unusual as menopause age can vary greatly among women. Medically, menopause is defined as the cessation of menses for 12 months and typically begins around the age of 51. However, the range is variable (45-55 years), and so it is also plausible that she is experiencing a normal physiological process. 3) CLINICAL REASONING: Considering that the patient does not have intermenstrual spotting, a symptom that would merit endometrial biopsy, there's little reason to suspect pathological changes in her uterine lining. Neither does she have any weight loss, bloating, urinary symptoms or pelvic pain, which would validate an indication for a pelvic ultrasound. Serum estradiol and follicle-stimulating hormone concentrations, being clear indicators for menopause, could have been useful if the patient had exhibited atypical symptoms or if there were concerns about induced menopause. On examining the options, recommending losing weight to facilitate the onset of menopause seems like a premature and unnecessary intervention as there's no clear evidence linking weight loss with triggering menopause. While being overweight may delay the onset of menopause, losing weight does not necessarily quicken its onset. Similarly, recommending leuprolide acetate injections is not suitable as it is a treatment for endometriosis and fibroids and can induce artificial menopause, which is not what we're aiming for. Furthermore, referral to an endocrinologist is not warranted at this stage as there's no sign of endocrine pathology. 4) CONCLUDING: Based on clinical reasoning and patient's history and presentation, it is most probably a case of delayed natural menopause. The absence of alarm symptoms like heavy or irregular menstruation, hot flushes, and night sweats combined with the family history of late menopause supports this conclusion. Clinically, it remains a normal variation to complete menopause beyond 55 years of age; this is due to the biological differences among women in the number of eggs they are born with and the rate at which these eggs are lost. 5) RESPONSE: Therefore, the most appropriate next step in managing this patient's concern about her continued menstruation would be to (D) Provide reassurance that she is simply still premenopausal, affirm that her continued menstruation is normal, and offer regular follow-ups to monitor any changes or occurrence of menopausal symptoms.

**Facilitator** **[Round 2]:** Summary: AI_A believes that the patient is likely still in her premenopausal phase due to the absence of menopausal signs and the presence of regular menses. They suggest that reassurance is the most appropriate next step. AI_B, on the other hand, recommends ordering serum estradiol and follicle-stimulating hormone concentrations, along with pelvic ultrasonography, to rule out any underlying endometrial pathology. They believe that the patient's age and obesity increase the risk of endometrial hyperplasia and carcinoma, which could contribute to continued menstruation. AI_C also recommends ordering serum estradiol and follicle-stimulating hormone concentrations to determine the patient's hormonal status and whether she is premenopausal or nearing the onset of menopause. AI_D, like AI_A, believes that the patient is simply still within the normal range of age for the onset of menopause and her continued menstruation does not warrant invasive procedures or hormonal tests. They suggest providing reassurance that she is simply still premenopausal. AI_E agrees with AI_A and AI_D, suggesting that the patient is likely experiencing a normal physiological process and that reassurance is the most appropriate next step. Question to clarify disagreements: Given the patient's age, family history, and lifestyle, what specific factors lead you to believe that further testing is necessary (for AI_B and AI_C) or not necessary (for AI_A, AI_D, and AI_E)? How do you weigh the potential risks and benefits of further testing versus reassurance in this case?. Based on your preceding response, how would you answer the original question: [A 55-year-old woman, gravida 1, para 1, comes to the office because she is concerned that she has continued to have regular menses while her friends of the same age have gone through menopause. She reports having her usual premenstrual breast tenderness and menstrual cramping. She has not had change in flow, intermenstrual spotting, hot flushes, or night sweats. Medical history is unremarkable. She takes no medications. She does not smoke cigarettes. She drinks one glass of wine each evening. Her last menstrual period occurred 3 weeks ago. Screening mammogram obtained 1 year ago and all previous Pap smears, including her most recent Pap smear 2 years ago, have shown no abnormalities. Her 50-year-old sister also has not gone through menopause. The patient does not exercise. She is 157 cm (5 ft 2 in) tall and weighs 80 kg (176 lb); BMI is 32 kg/m². Vital signs are normal. Physical examination discloses adiposity of the abdomen. Pelvic examination discloses pink and rugose vaginal walls and a small uterus. The remainder of the physical examination discloses no abnormalities. Which of the following is the most appropriate next step to address the patient's continued menstruation? (A) Order fine-needle biopsy of the endometrium (B) Order pelvic ultrasonography (C) Order serum estradiol and follicle-stimulating hormone concentrations (D) Provide reassurance that she is simply still premenopausal (E) Recommend losing weight to help facilitate the onset of menopause (F) Recommend monthly leuprolide acetate injections for a 3-month duration (G) Refer the patient to an endocrinologist]

**AI_B:** Restating the question: The query seeks the best immediate next step in addressing a 55-year-old woman’s concern about her continued menstruation while her peers have commenced menopause. She has no signs typically indicative of menopause such as change in menses flow, spotting between menses, hot flushes, or night sweats. Moreover, a clinical check exhibits no irregularities. Planning: Assumptions in this case include that the patient might still be in her premenopause considering she does not present menopausal symptoms such as hot flushes or night sweats and that she has a family history of late menopause. Alternatively, her obesity could be contributing to her late menopause; or she might have an underlying endocrine abnormality maintaining her regular menstrual cycle despite her age. Clinical Reasoning: Menopause refers to the time of life when a woman naturally ceases her menses, which can vary among women, but typically it's around the early 50s. In this case, the woman, aged 55, continues to have regular menses with no other associated menopausal signs. The most common age range for menopause in women in Western countries is 48-55 years. Thus, the woman's continued menstruation isn't out of the ordinary. Additionally, her pelvic examination is unremarkable, showing normal vaginal walls and a small uterus. Concluding: The woman’s concern arises from her perception of her continued menstruation as anomalous in comparison to her peers who have started menopause. Given her late menopause relative to the average range age but still within a plausible range for menopause onset and her normal physical examination results, the absence of any menopausal symptoms suggests that she is still in the premenopausal stage. Response: Therefore, the most appropriate next step would be (D) to provide reassurance that she is still premenopausal. It's important to explain to her that menopause can occur at different ages in different women and that it's not startling that she hasn't reached menopause yet. Her regular menstrual cycle and lack of menopausal symptoms indicate that she is probably still premenopausal. Medical interventions or further testing are not currently warranted.

**AI_A:** Restating the question: This middle-aged woman is concerned about her continued menstruations while her age-related peers are going through menopause. She shows no signs of menopause, and she has no changes in her menstrual flow or abnormal symptoms. All her previous screenings have come back normal and she has a family history of late menopause. The need is to identify the most appropriate next step to address her concern. Planning: In this circumstance, we would need to consider several factors: her age, her health condition and lifestyle, her family history of late menopause, and absence of menopausal symptoms. While her BMI is above the optimal range, there is no indication of other health issues. She drinks moderately but does not exercise. Her continued menstruation could be due to a number of causes. Her premenopausal status could be genetically determined, or influenced by lifestyle factors. There's also possibility of some underlying issues riding on the fact that obesity can increase the risk of endometrial hyperplasia and carcinoma which could delay menopause. Clinical Reasoning: Since there are no suspicions of any menstrual irregularities or abnormalities it is unlikely that this case is an exception to the menses onset variations seen in many women. Given her family history of later menopause onset, genetic factors are a probable explanation for her continued menses. Despite being overweight, she is not showing symptoms of polycystic ovary syndrome or other conditions that might cause menstrual irregularities. She's also not reporting any symptoms related to possible menopausal transition such as hot flashes or night sweats. Concluding: Considering these factors, it may just be that this woman's body is taking longer to reach menopause relative to her peers. It would be premature to immediately assume an underlying pathology without further investigation. Invasive procedures like endometrial biopsy or hormone therapy would be unwarranted given the current facts. On the other hand, hormonal assays may be of interest to corroborate the premenopausal status, but that would only be needed if she presented with symptoms suggesting early menopause or a disorder affecting her reproductive system. So, reassurance may be the more appropriate next step. Response: Based on a comprehensive review of the presenting case, this patient is likely premenopausal and it's normal to have variations in menopausal onset among women. While the hormonal statuses to confirm the possibility of menopause could be considered if there were abnormal symptoms of early menopause, given the current scenario, I would support the option "(D) Provide reassurance that she is simply still premenopausal" as the most appropriate step to address this patient's continued menstruations.

**AI_C:** The question essentially inquires about the appropriate management step for a 55-year-old woman who continues to menstruate despite being in the age category where menopause usually occurs. Menopause, which signifies the end of woman's reproductive years, is characterized by the cessation of menstruation for more than 12 months and is associated with a decline in the production of hormones, such as estrogen and progesterone. Generally, menopause happens between the ages of 45 and 55, but the timing can vary significantly. Moving forward, we have two prominent assumptions: One being that the woman is simply a late bloomer to the menopausal process, and second that her condition could be a sign of an underlying endocrine or gynecologic condition that's preventing her from attaining menopause. However, considering the absence of any worrisome findings in her physical examination or past medical history suggestive of endometrial pathology or an endocrine disorder, the normal physiological process of menopause, although delayed, is a necessary assumption to prioritize. Clinical reasoning leads us to mull over the concept of late menopause, which is not uncommon and can be influenced by various factors including family history, which is evident in the patient's case as her sister too hasn't gone through menopause yet. Furthermore, there's no talk about typical menopausal symptoms including night sweats, hot flashes or vaginal dryness which could hint towards a nearing menopause. Rather, her regular menstrual cycles and symptoms, and lack of any abnormal medical history relay a different story altogether. As we segway into concluding, we can affirm that probable late menopause, backed by genetic influence seems to be the case for our patient. No evidences pointing towards an endocrine disorder or endometrial pathology calls for no invasive steps or referrals for now. The fact that she is worried about her continued menstruation is where the role of the physician's reassurance comes into play, letting her know that late menopause is a possibility and is nothing to be worried about until any abnormal signs start to show up. Therefore, in response, providing the woman assurance that she is likely premenopausal and there's no need to worry would be the most suitable option. It's a normal process and the timeline can sway on either side depending on various contributing factors. So the answer would be - (D) Provide reassurance that she is simply still premenopausal.

**AI_E:** Restating the question: A 55-year-old woman, gravida 1, para 1, comes to the clinic as she is worried about her regular menstrual cycle while her peers of the same age have hit menopause. She is experiencing her usual monthly menstrual symptoms, with no changes in flow or signs of menopause such as hot flushes or night sweats. The patient's medical history presents no outstanding issues and she takes no medication. Although she consumes alcohol daily, she does not smoke and her physical examination is normal. Her family history reveals that her sister, who is 50 years old, also has not reached menopause. The patient doesn't engage in any physical exercise and her BMI shows that she is obese. We are asked to determine the most appropriate next step addressing her continued menstruation. Planning: Before hypothesizing on the most suitable way to handle the patient's situation, it is crucial to consider a few assumptions. One primary assumption is that this patient is experiencing a delayed onset of menopause due to her genetic predisposition, with her sister also reporting delayed menopause. Another potential assumption could be that the continued menstruation might be due to her obesity, which could alter hormone production and delay menopause. Lastly, we could consider that the continued menstruation is due to an underlying pathological condition. Clinical Reasoning: The clinical thought process would begin by exploring the probability of each assumption stated above. Evaluating the patient's family history would provide a broader perspective on whether her continued menstruation could be due to a genetic predisposition. Additionally, considering her BMI, one should evaluate if weight loss would help her hit menopause. Lastly, ruling out pathological causes could offer a comprehensive approach to determine the source of her continued menstruation. Concluding: After careful evaluation, the genetic predisposition seems most plausible due to her sister's similar experience. Her obesity might contribute to the delay, but seeing as her menses is regular and she doesn't exhibit signs of menopause, it might just be that she's experiencing a normal physiological process slightly later than some of her peers. Without any alarming signs or symptoms, immediate invasive investigations might not be the best course of action. Response: In response to the question, option D - Provide reassurance that she is simply still premenopausal seems the most appropriate course of action. It is not uncommon for women to experience menopause at different ages and her symptoms do not suggest any pathological conditions. However, regular monitoring, coupled with encouraging a healthier lifestyle, might be a suitable approach for now.

**AI_D:** Restating the Question: The primary concern here is a woman of 55 years who is still experiencing her regular menstrual cycle while her peers have already undergone menopause. She has a history of being overweight, enjoying nightly glasses of wine, and not regularly exercising. There has been no change in the character of her menstrual cycle, and she displays typical signs of premenstrual breast tenderness and menstrual cramping. Apart from an overweight body mass index (BMI) and lack of physical activity, her medical history and physical exam reveal no significant findings. The choice lies between systems of action to assess her physiological status, with choices such as hormone testing, pelvic sonography, endometrium biopsy, and referral to an endocrinologist, or reassurance that she is within normal parameters and continued menstruation at her age is not unusual. Planning: The primary assumption in this case is that the patient's continued menstruation is a normal phenotype of menopause onset and merely delayed. An alternate assumption could be that her endocrine system might be affected by internal or external factors or underlying pathological conditions influencing her continued menstrual cycle. When assessing these assumptions, the clinical priority should include assessing the woman's hormonal status and ruling out the possibility of any inherent pathologies affecting the endocrine system, which could lead to persistent menstruation. Clinical Reasoning: The patient's age does suggest a possible onset of menopause, but it's not uncommon for women to have their first menopausal symptoms at different ages, depending on various factors such as genetics, lifestyle, or overall health status. In a clinical setting, it's essential to appreciate the natural variety in menopause onset ages and to understand the potential implications of a late menopause onset. The fact that the patient's sister has also not undergone menopause could suggest a genetic predisposition towards a later menopause. Concluding: Based on the patient's age, symptoms, and family history, it is plausible to assume that she is still in her late stages of reproductive life. Her menstrual cycle's regularity and the absence of other menopausal symptoms, such as hot flashes or night sweats, indicates an active reproductive hormonal axis. Her obstetric history and the absence of abnormal findings in her annual mammogram and Pap smears suggest a low likelihood of underlying pathological abnormalities. However, her increased BMI could have slight implications on her hormonal balance, possibly causing a delay in menopause onset. Response: Given all the considerations, I concur with AI_A, AI_D, and AI_E that reassurance is the most appropriate next step in addressing this patient's continued menstruation. Explaining to her that there can be variation in the timing of menopause onset could alleviate her worries. This would obviate the need for an invasive procedure and reduce the potential risk and inconvenience associated with unnecessary hormonal tests. (Answer D)

**Facilitator** The consensus among the AI responses is that the most appropriate next step to address the patient's continued menstruation is to provide reassurance that she is simply still premenopausal (Option D). This conclusion is based on several factors. Firstly, the patient's age, health condition, lifestyle, and family history of late menopause are considered. The patient shows no signs of menopause, such as changes in menstrual flow or abnormal symptoms, and all her previous screenings have come back normal. Secondly, the patient's continued menstruation could be due to a number of causes, including genetic factors or lifestyle influences. However, there are no suspicions of any menstrual irregularities or abnormalities, making it unlikely that this case is an exception to the variations seen in many women. Lastly, considering the patient's age, symptoms, and family history, it is plausible to assume that she is still in her late stages of reproductive life. Her regular menstrual cycle and the absence of other menopausal symptoms indicate an active reproductive hormonal axis. Therefore, the consensus is that reassurance is the most appropriate next step in addressing this patient's continued menstruation.
